# Unveiling the intricacies of cardiac valve pathophysiology

**DOI:** 10.3389/fcvm.2025.1570271

**Published:** 2025-07-24

**Authors:** Johannes H. Jedrzejczyk, Oline Hjertensgaard, Victor G. Puelles, J. Michael Hasenkam

**Affiliations:** ^1^ Department of Cardiothoracic and Vascular Surgery, Aarhus University Hospital, Aarhus, Denmark; ^2^ Department of Clinical Medicine, Aarhus University, Aarhus, Denmark; ^3^ Department of Pathology, Aarhus University Hospital, Aarhus, Denmark; ^4^ III. Department of Medicine, University Medical Centre Hamburg-Eppendorf, Hamburg, Germany

**Keywords:** valvular heart disease, cardiac valves, cardiac valve physiology, cardiac valve pathophysiology, valve endothelial cells, valve interstitial cells

## Abstract

**Introduction:**

Heart valves have long been regarded as uncomplicated, avascular, and passive structures. However, we hypothesise that their structure and function are complex. Therefore, we have reviewed the available literature to gain a profound understanding of their cellular composition and (patho)physiological behaviour.

**Methods:**

A systematic search for articles related to the anatomy, histology, and physiology of heart valves was conducted using PubMed and Google Scholar, as well as a manual search of journals and websites. All publications were screened by title and abstract, and potentially eligible articles were reviewed in full text to assess their relevance.

**Results:**

Cardiac valves comprise a complex, three-layered structure composed of an intricate network of cells. Valvular endothelial cells cover the atrial and ventricular sides of the valves. Valvular endothelial cells are morphologically and functionally distinct from vascular endothelial cells and play a crucial role in maintaining valve function. The three-valve layers, lamina fibrosa, spongiosa, and ventricularis, exhibit distinct biomechanical properties due to their varying extracellular matrix components and valvular interstitial cells. Valvular interstitial cells can be divided into four subtypes, each exhibiting specific cellular functions essential for normal valve physiology. However, pathological stimuli can cause aberrant activation of the valvular interstitial cells, leading to valve calcification and stenosis.

**Conclusion:**

The intricate interplay of cellular components within cardiac valves is vital for maintaining normal valve function and structural integrity, but also contributes to valve pathology. A holistic understanding of heart valves, integrating cellular, molecular, and neural perspectives, is needed in the future.

## 1 Introduction

Valvular heart disease is a significant contributor to cardiovascular morbidity and mortality on a global scale (1). The disease burden associated with valvular heart disease is expected to rise in the upcoming decades due to an ageing population and the growing prevalence of multiple risk factors, including hypertension and diabetes. Medication can help alleviate symptoms and delay the progression of the disease (2–4). However, as the disease advances, surgical intervention is often required to repair or replace a damaged heart valve (2–4). While heart valves were previously believed to be passive structures that do not play an active role in maintaining function, tissue integrity, and durability, recent scientific evidence suggests that these structures are far more complex than previously thought. Although the opening and closure of valves are triggered by changes in transvalvular pressure during the cardiac cycle, valves are not entirely passive structures; their active role in cardiovascular physiology and pathophysiology is more complex than previously assumed (5). Therefore, it is crucial to gain a deep understanding of the physiology and structure of heart valves to enhance treatment and improve outcomes for the large number of patients with valvular heart disease.

**Figure 1:**
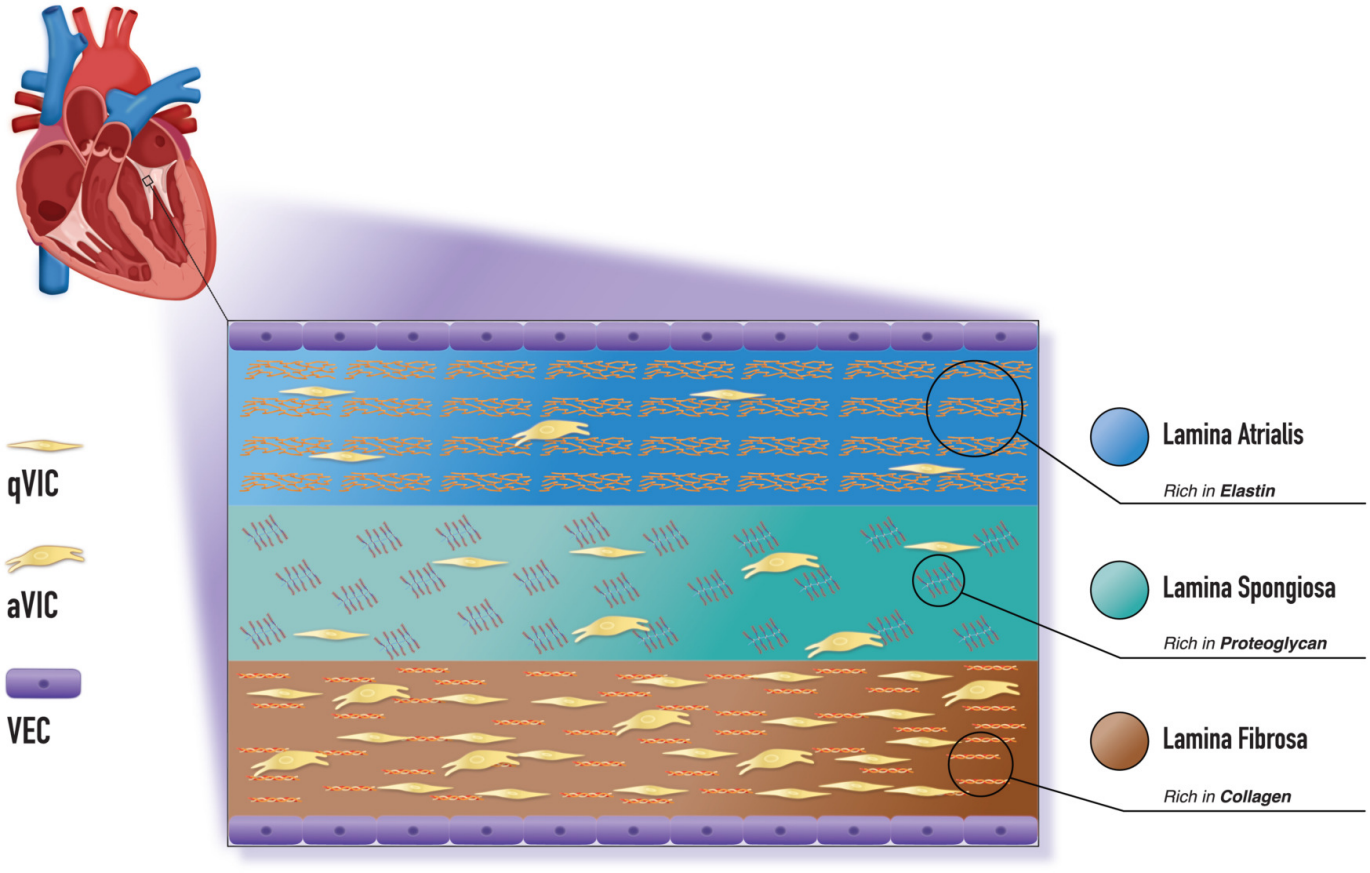
A depiction of the histological structure of the mitral valve with three distinct layers: the lamina atrialis, lamina spongiosa, and lamina fibrosa, along with the cellular elements comprising each layer. aVIC, activated myofibroblast-like valve interstitial cells; VEC, valve endothelial cell, qVIC, quiescent fibroblast-like valve interstitial cell.

Regardless of anatomical position, heart valves are composed of leaflets attached to a fibrous ring. Each leaflet comprises a thick extracellular matrix organised in three distinct layers—lamina fibrosa, spongiosa, and ventricularis, as shown in Figure 1. During the early stages of embryonic heart development, the heart begins to form as a tube consisting of two layers: the endocardium and the myocardium, separated by an extracellular matrix known as cardiac jelly (6). Endothelial cells from the endocardium are activated by TGF-β2 and TGF-β3, transforming into a subset of mesenchymal cells that can migrate into the cardiac jelly and develop into mature valves. This process, known as endothelial-to-mesenchymal transformation (7, 8), is crucial to the unique matrix compositions in the three distinct valve layers, which give them their distinct mechanical properties. The lamina atrialis is abundant in elastin, an essential component that facilitates tissue recoil during systolic and diastolic conformation. Lamina spongiosa is the softest layer due to the large amounts of proteoglycans and numerous cells, including the valve interstitial cells (VICs). Finally, the lamina fibrosa comprises a dense network of anisotropically arranged collagen bundles, providing strength and flexibility to the valve areas exposed to the maximum diastolic stress, such as the edges and the “belly” (9). The three distinct layers of the heart valve—the fibrosa, spongiosa, and ventricularis—work synchronously to facilitate the smooth opening and closing of the valve.

Valve endothelial cells (VECs) are a distinct population of endothelial cells that cover the inside of the heart and its valves, possessing unique properties not observed elsewhere in the vasculature (10). In addition, VICs have been discovered in the lamina spongiosa, and their structure and function are far more complex than previously believed. The discovery of these diverse cell types in heart valves raises the question of whether the cellular components of cardiac valves are more intricate than previously thought. Additionally, a recent study has identified the presence of distinct nerve terminals in heart valves and a close association of varicose nerve fibres with endothelial, fibroblast, and smooth muscle cells (11), suggesting that neural involvement may play a role in regulating valvular function.

Changes in the cellular components of the heart valve are associated with valvular heart disease. Alterations of the extracellular matrix in the cardiac valves can be caused by inflammation resulting from various stressors, such as hyperlipidemia and smoking (10). This inflammatory process can lead to the accumulation of lipids, which stimulate interstitial cells to adopt a matrix pro-remodelling phenotype, potentially causing tissue calcification (12), altering the overall mechanical properties of the valve (13). Given the complex processes involved in health and disease, a thorough understanding of the individual components that contribute to cardiac valve diseases is crucial.

Heart valve pathologies are often treated with surgical interventions that focus on implanting a passive artificial material to secure immediate valve functionality. However, this approach overlooks the importance of the complex valve structures and functions. To minimise the complications associated with biological and mechanical valve replacement, a more comprehensive approach is necessary in the future. This review aims to discuss the intricacies of heart valves and provide a more sophisticated understanding of their cellular structure and unique function. This knowledge is particularly relevant for developing tissue-engineered heart valves, which aim to create a native replica encompassing a distinct cellular structure and unique function.

## 2 Materials and methods

This literature review was conducted using a systematic approach. PubMed and Google Scholar were searched for relevant articles from May 1, 2024, to July 9, 2024, with the language restriction set to English.

### 2.1 Search strategy and study collection

A systematic approach was used to assess all publications. Starting with the controlled subject heading “heart valve.” Controlled subject headings were used to search for data on the following terms: “cytology,” “physiology,” “histology,” “structure,” and “pathophysiology.” Subsequently, a second search was performed using the search words “endothelial cells,” “interstitial cells,” “endothelial-to-mesenchymal transition,” “VIC quiescent,” “VIC active,” “osteoblast-like,” “neurons,” which were related to the findings in the main articles. All articles were screened by title and abstract, and potentially eligible articles were read in full text to evaluate their relevancy. In addition to database searches, relevant articles were manually searched in journals and websites, and reference searches were conducted in the bibliographies of these articles.

### 2.2 Eligibility criteria

Inclusion criteria: articles published in peer-reviewed journals; studies reporting original data relevant to heart valve biology, structure, function, or biomechanics; publications focusing on endothelial cells, valvular interstitial cells, or their transitions and phenotypes; studies employing either *in vivo*, *in vitro* or computational models; human, porcine, sheep or rodent valve tissue; full-text articles. Exclusion criteria: articles not published in English; review articles; conference abstracts; editorials with no primary data; studies focusing on cell types unrelated to cardiac valves; valve tissue from species other than human, porcine, sheep, or rodent; articles with insufficient methodological details to assess quality.

### 2.3 Data collection

In each study, the following information was gathered: author, country of study, year of publication, journal of publication, and study design.

## 3 Results

**Figure 2:**
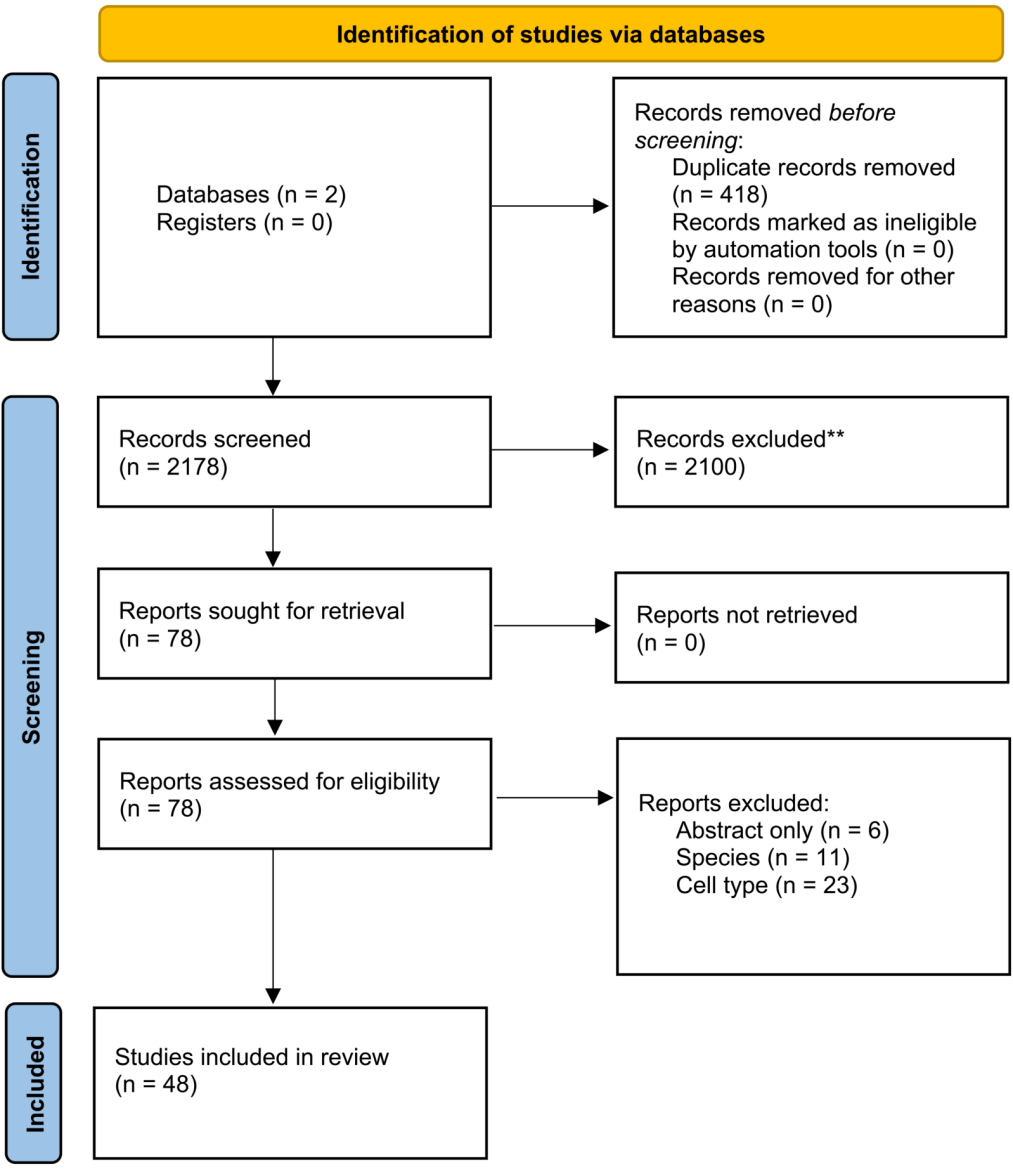
PRISMA flow chart used for study selection.

We identified 2.596 studies. After screening for title and abstract and removing duplicate studies, 78 studies were considered eligible for full-text assessment. Forty-eight articles were used to synthesise this review (Figure 2).

### 3.1 Valvular endothelial cells

VECs cover the entire surface of the valve and act as a barrier between the blood components and the underlying valve tissue. These cells exhibit unique morphological characteristics that differ from vascular endothelial cells. Under static *in vitro* conditions, VECs assume a more stellate pattern than vascular endothelial cells, reflected by a lower shape index (0.78 ± 0.08 vs. 0.84 ± 0.07) (14). Vascular endothelial cells exhibit an elongated structure and alignment in high-shear stress conditions, parallel to their axis. Interestingly, a study by Miragoli et al. demonstrated that vascular endothelial cells on the aortic aspect of the aortic valve cusps showed greater alignment than those on the ventricular surface of the valve, which experiences higher shear stress (15). This suggests that the morphology and alignment of VECs may be more influenced by pressure stress than shear stress. Although canine aortic VECs and cultured porcine aortic VECs orient themselves perpendicular to the flow direction rather than parallel (14, 16), human-cultured aortic VECs do not, indicating that VEC alignment may vary based on the species (17).

The arrangement of VECs can significantly impact compliance. Miragoli et al. demonstrated that VECs on the ventricular side of the aortic valve are notably less compliant than those on the aortic side, which are more aligned along a single axis. This difference in compliance was observed even when VECs from the two surfaces of the aortic valve were selectively cultured. Hence, the differences in compliance cannot be solely attributed to differences in the stiffness of the underlying tissue. Additionally, the fact that these differences were observed in cultured cells not exposed to flow suggests that the difference in compliance is not solely due to different surface flow patterns. Instead, Miragoli et al. proposed that epigenetic pathways regulating gene expression, such as NO type III, may confer the differences to the VECs on either side of the valve, which determine their mechanical properties (15).

The transformation of endothelial cells to mesenchymal cells (endothelial-to-mesenchymal transformation) plays a significant role during embryonic development. Pioneering research has demonstrated that during embryonic development, the transition of endocardial cells into cardiomyocytes initiates biosynthetic processes, culminating in the formation of cardiac cushion mesenchyme and cardiac valves (18, 19). Endothelial-to-mesenchymal transformation can also occur in adulthood and can be induced by factors such as oxidative stress, fatty acid oxidation, hypoxia, hyperglycemia, and shear stress (20). Furthermore, it has been demonstrated that endothelial-to-mesenchymal transformation in heart valves can be potentiated by exposure to cyclic mechanical strain (21). VECs in adult heart valves can undergo endothelial-to-mesenchymal transformation (22, 23) and may play a role in the pathogenesis of cardiovascular disease in adulthood, such as calcific aortic valve disease (22, 24, 25). VEC dysfunction has been identified as an essential factor in the early stages of calcific aortic valve disease. This dysfunction has been linked to the recruitment of immune cells (26), disruption of protective nitric oxide signalling (27, 28), and the ability of VECs to change their characteristics and express proteins that contribute to calcification (29). These findings suggest that dysfunctional VECs may play a significant role in the development of valvular disease.

Nevertheless, VICs have been shown to play a significant role in regulating VECs. Hjortnaes et al. found that VICs have an inhibitory effect on endothelial-to-mesenchymal transformation and VEC osteogenesis. However, VECs did not inhibit VIC mineralisation. Time course analysis led the authors to conclude that endothelial-to-mesenchymal transformation precedes VEC osteogenesis. This study highlights the importance of VEC-VIC interactions in maintaining valve homeostasis (22).

It has also been suggested that endothelial-to-mesenchymal transformation plays a role in the pathogenesis of mitral valve disease. Mitral VECs have been observed to differentiate into mesenchymal cells, with some demonstrating osteogenic potential *in vitro* (29, 30). It has, therefore, been proposed that endothelial-to-mesenchymal transformation may contribute to the renewal of VICs. Moreover, the VECs may also exhibit osteogenic characteristics under pathological conditions and could induce valvular calcification (29, 30). A related study has explored the potential role of endothelial-to-mesenchymal transformation in the mitral valve changes associated with valve prolapse. This study identified elevated circulating osteoprotegerin levels in patients undergoing mitral valve replacement due to valve prolapse. Using cultured VECs isolated from the pathological mitral valves, they later found that osteoprotegerin, primarily associated with bone metabolism, can induce the endothelial-to-mesenchymal transformation process. Notably, the study also revealed a novel discovery: that mitral valve endothelial cells undergoing endothelial-to-mesenchymal transformation were capable of producing and secreting osteoprotegerin. This finding suggests the existence of an autocrine pathogenic mechanism for endothelial-to-mesenchymal transformation (31).

### 3.2 Valvular interstitial cells

**Figure 3:**
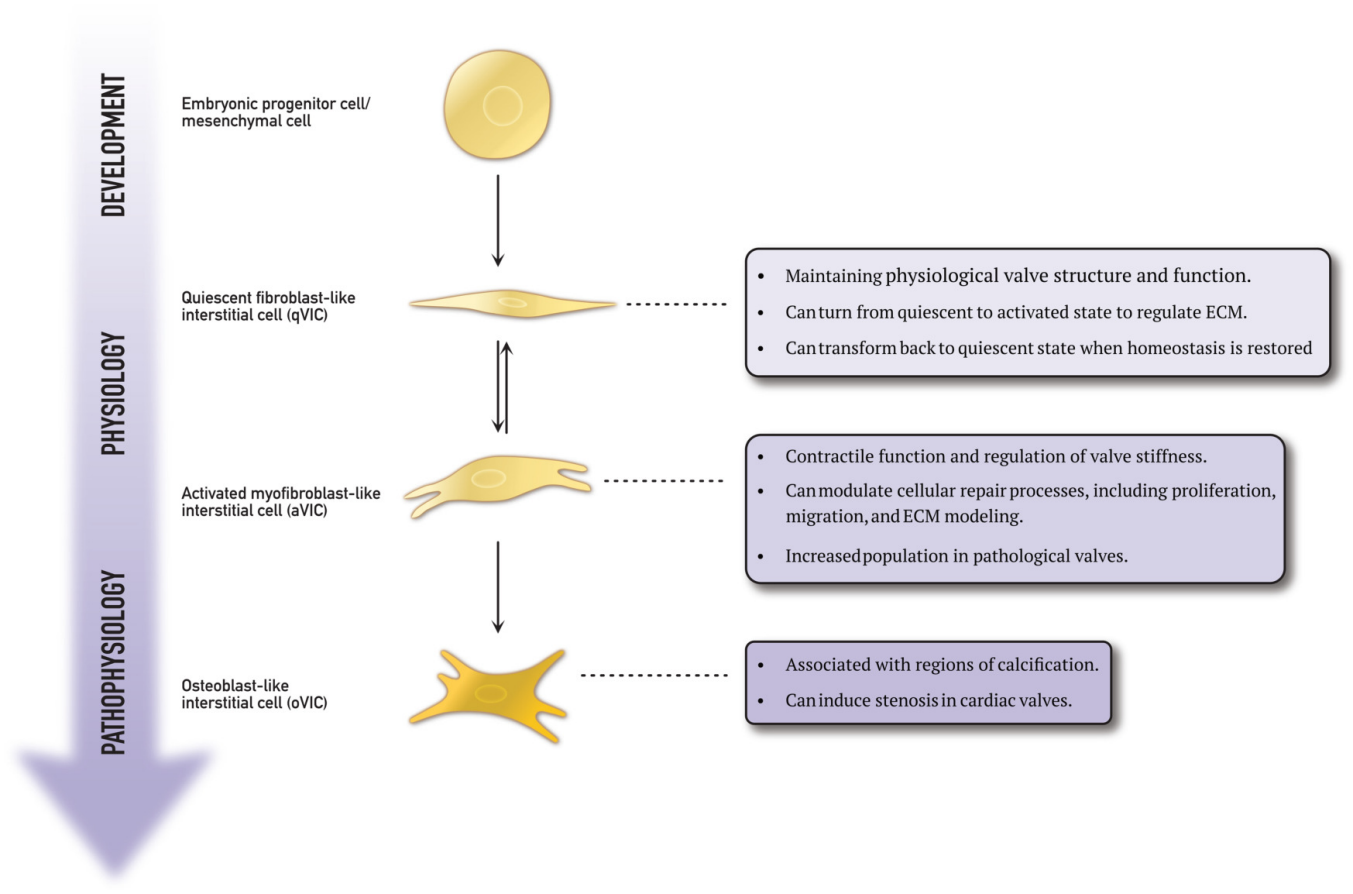
This schematic representation explains the differentiation process of embryonic progenitor cells and mesenchymal cells into various valvular interstitial cell phenotypes.

VICs are the most prevalent cells in the heart valves and are found in all three layers of the cusps. Rippel et al. determined the densest concentrations of VICs to be in the lamina fibrosa (32). A study by Butcher et al. revealed that VICs possess contractile properties and the ability to synthesise matrix components (33). Based on these findings, the authors suggested that VICs play a crucial role in maintaining the leaflet environment (33). Various VIC phenotypes have been identified in diseased human heart valves, each exhibiting a specific set of cellular functions essential in normal valve physiology and pathological processes. Figure 3 schematically shows the four phenotypes.

As previously mentioned, embryonic progenitor and mesenchymal cells play a crucial role during the endothelial-to-mesenchymal transformation during embryogenesis. This process is also implicated in the development of cardiovascular disease (22, 24, 25). Wang et al. discovered a group of VICs expressing markers characteristic of progenitor cells in porcine heart valves (34). These cells were located near the centre of the valve. Progenitor cells are present in many tissues, such as the bone marrow and circulation, and can enter the valve (35, 36). Chen et al. identified a subpopulation of multipotent mesenchymal progenitor cells in aortic VICs (37). The multilineage potential of both freshly isolated and subcultured aortic VICs was evaluated *in vitro*. The study found that the VICs demonstrated inducibility to osteogenic, adipogenic, chondrogenic, and myofibrogenic lineages. Moreover, a high-frequency putative osteoprogenitor subpopulation was identified within the VIC population. These morphologically distinct cells were shown to self-renew and produce bone matrix from single cells. This study demonstrates the presence of a mesenchymal progenitor cell population within the aortic valve, with the potential to significantly contribute to valve calcification (37).

Quiescent fibroblast-like valvular interstitial cells (qVICs) are dormant in the mature adult heart valve. Their role is to maintain the physiological valve structure and function (38). Once stimulated, qVICs are reprogrammed to differentiate into activated myofibroblast-like VICs (aVICs) to regulate the functional remodelling of the extracellular matrix collagen (25). Rabkin et al. discovered that aVICs can return to quiescence when equilibrium is restored (38). This dynamic transformation between quiescent and active states is vital in sustaining normal valve function. It has been demonstrated that pathological stimuli can also activate qVICs (39). During their differentiation from qVICs to aVICs, the phenotype and morphology change from spindle/tailed to rhomboid cells (40). The qVIC phenotype is known to be unstable and difficult to study (41). However, Roosens et al. attempted to investigate this by creating a 3D *in vitro* model of aggregated VICs (42). They successfully preserved the quiescent/native state of the VICs by down-regulating α-smooth muscle actin expression. The VIC aggregates were able to produce their own extracellular matrix, resembling native valve composition.

aVICs have been found to express the same contractile proteins and transcription factors as those found in skeletal muscle (43). However, they also express α-smooth muscle actin, a marker commonly found in myofibroblasts (44). In addition to *α*-smooth muscle actin, aVICs also express other cell markers, such as embryonic non-muscle myosin heavy chain and TGF-β, typically not found in qVICs (40, 45). When activated, VICs have two key functional properties. First, their myogenic structure allows them to contract, and second, they can synthesize extracellular matrix components. The contraction of aVICs has been demonstrated to alter the mechanical properties of valve tissue. El-Hamamsy et al. have observed that increased aVIC contraction leads to an increase in valve stiffness and that various vasoactive agents can enhance intracellular calcium levels and, thus, the degree of aVIC contraction (46).

Additionally, endothelial cells appear to be synthesising these vasoactive agents. Ku et al. discovered that the property of contraction impacts not only the structure of the valve but also the synthesis of collagen (47). They observed that the degree and duration of stretch applied to cells and tissue affect collagen synthesis.

The role of activated VICs in valvular disease has been studied extensively. In particular, Rabkin et al. investigated human mitral valves taken from patients with myxomatous degeneration and compared them to native mitral valves (45). They found that VICs in myxomatous valves exhibited activated myofibroblast-like features and expressed excessive levels of catabolic enzymes. Numerous studies have identified a subtype of VICs called osteoblast-like VICs (oVICs). These cells exhibit a distinct cellular phenotype and produce a more calcified matrix under osteogenic conditions than other VICs (34). oVICs can deposit calcium in the extracellular matrix, leading to stenosis (48).

### 3.3 Neurons

The primary sensory and autonomic components of the nervous system play a crucial role in regulating the heart valve function. Marron et al. examined the density and distribution of nervous innovation in heart valves using confocal microscopy, histochemical, and quantitative immunohistochemical techniques (11). In the atrioventricular valves, the nerves are situated in the atrial aspect of the valve and extend over the proximal and medial portions of the leaflets. Conversely, the nerve fibres in the arterial valves are situated on the ventricular aspect of the valve. Variations in innervation were also observed within the atrioventricular valves and arterial valves. In the mitral valve, Maroon et al. discovered a two-fold higher nerve density within the anterior mitral valve leaflet compared to the posterior leaflet. Although the arterial valves generally have a similar density of innervation, the noncoronary leaflet of the aortic valve has reduced innervation compared to the two coronary leaflets. The study also revealed that no nerves reached the free edges of the leaflets or the fibrous core in all four heart valves. Finally, Marron et al. discovered that nerve fibres were closely related to VECs and VICs. Similarly, Steele et al. investigated intrinsic cardiac neurons in guinea pig hearts (49). The researchers observed that the most concentrated innervation by axons of intrinsic neurons within the heart occurred in the valves. The findings emphasise the complex and varied innervation patterns of heart valves, underscoring their significance in valve function. A detailed understanding of neural distribution may change the future therapeutic strategies for heart valve diseases.

## 4 Discussion

This review of primary research articles examines the intricate cellular and neural mechanisms underlying heart valve function. The predominance of *in vitro* studies using animal tissues underscores the challenges in replicating the complex *in vivo* environment of human valves. Nonetheless, the transition to *in vivo*-like studies and 3D modelling represents a significant advancement in closely mimicking physiological conditions. Due to the limited number of studies on human tissue, this review includes studies conducted on both human tissue and tissue from other mammalian species. Thus, the question of whether it is sound to compare cardiac valve tissue across species quickly arises. Although the porcine species are known to share remarkable similarities with their human counterparts in terms of cardiac anatomy and physiology (50), studies have identified differences in the extracellular matrix composition between the two species (51). As such, using animal tissue and cells for research always represents uncertainties due to interspecies differences. However, animal tissue studies remain a viable alternative due to the ethical and practical constraints of using human tissue and cells.

VECs exhibit distinctive morphological characteristics influenced by various mechanical stressors, particularly shear and pressure. Studies, including those by Miragoli et al., reveal that the alignment and compliance of VECs are not solely determined by shear stress but are also influenced by intrinsic factors that epigenetic pathways may regulate (15). Thus, VECs can exhibit epigenetic signatures reflective of their tissue of origin, suggesting a potential for retaining their phenotype. However, the long-term stability of these signatures during extended culture remains uncertain, and endothelial plasticity is known to be influenced by environmental factors (52). In most cases, cultured endothelial cells tend to adopt a more uniform phenotype, highlighting the importance of local signalling and biomechanical cues (53). This plasticity poses both challenges and opportunities in engineering donor-specific, biocompatible heart valve substitutes. Understanding the extent to which VEC phenotypes can be preserved or reprogrammed in engineered scaffolds remains a critical frontier in valvular tissue engineering. This nuanced comprehension of VEC behaviour underscores their vital role in maintaining valve integrity and function. Moreover, the phenomenon of endothelial-to-mesenchymal transformation in VECs during development and adulthood suggests its potential contribution to valve pathology, including calcific aortic valve disease and mitral valve prolapse. The interactions between VECs and VICs are crucial in modulating these processes, indicating that therapeutic interventions targeting these interactions could be beneficial in preventing or treating valvular diseases.

It is well established that the mesenchymal cells are responsible for the embryonic origin of VECs and VICs (6, 7, 36). However, the maintenance of these cells through ageing and the variation in their numbers over time have yet to be adequately investigated. While some studies have focused on the health and disease of the valves, few have investigated valves from different age groups. Anstine et al. studied various cell markers across embryonic to adult stages and found an increased percentage of cells expressing hematopoietic markers (54). This finding suggests an increase in the number of extracardiac cells in the valves as age progresses. However, a better understanding of the changes in the cell population over time is needed.

VICs, the predominant cells in heart valves, are pivotal in preserving and reorganising the extracellular matrix. Their dynamic shift between quiescent and activated states, as well as their potential differentiation into osteoblast-like cells, emphasises their involvement in normal valve function and pathological calcification. Identifying progenitor and mesenchymal cells within the VIC population adds another layer of complexity, suggesting a potential source for regenerative therapies. The potential of VICs in regenerative therapies offers promise for the future of treatment for valve diseases.

Although valvular calcification and atherosclerosis share several pathophysiological mechanisms and risk factors, this overlap has not translated into equivalent therapeutic responses. Notably, statins, while effective in reducing the atherosclerotic burden, have consistently failed to demonstrate a clinical benefit in slowing the progression of calcific aortic stenosis in randomised controlled trials (55). This is despite *in vitro* studies showing that statins can attenuate osteogenic differentiation and calcification in cultured VICs (56). The reasons for this discrepancy remain unclear but may relate to differences in local microenvironments, disease stage at treatment initiation, or pharmacokinetic limitations in targeting valve tissue. These findings underscore the need for valvular-specific therapeutic strategies rather than extrapolation from vascular disease paradigms.

The intricate innervation of heart valves, detailed by Marron et al. (11) and Steele et al. (49), underscores the influence of the nervous system on valve function. The varying distribution of nerve fibres in different valve regions indicates that innervation may impact the mechanical and functional properties of the valves. Understanding these neural patterns not only presents an intriguing prospect but also holds the potential to lead to innovative therapeutic approaches that capitalise on the nervous system to maintain or restore valve function.

Developing three-dimensional *in vitro* models, such as organoids, is crucial in understanding the cells in adjacent tissues. However, numerous challenges need to be addressed. For instance, VICs must be preserved and recovered before being studied in an actual valve model (42). To create an accurate 3D model, VICs must form an aggregate to mimic the *in vivo* microenvironment. In tissue engineering, ongoing exploration of new hypotheses aims to create cardiac valves that closely resemble native valves and can be used in heart surgery. A combination of *in vivo* and *in vitro* studies can enhance the understanding of the complexity of cellular components. *in vitro* studies on the cells in the valves have provided valuable insights into their components; however, these findings should be considered in the context of the cells’ functions, including communication through mediators and cellular pathways. An examination of the subject from multiple perspectives can provide a more comprehensive understanding of the native heart valve, which can contribute to the advancement of research into valve diseases and the development of surgical treatments.

Heart valves are currently treated from a purely mechanical perspective, with little consideration for their biological function. The diseased valve is typically replaced with a passive mechanical structure, but this review has revealed that this approach may be oversimplified. Heart valves are incredibly complex, with a dynamic cellular composition enabling them to perform various functions. To truly understand and treat diseased heart valves, we need a more nuanced approach considering the biological factors at play. Bioscaffolds offer a promising avenue for research, enabling the body to rebuild full valve function with an active, dynamic cellular composition that mimics the complexity of genuine heart valves. In this context, it is noteworthy that heart valves have been proposed to exhibit a degree of immune privilege, attributed to their avascularity, limited regenerative capacity, and low expression of MHC and ABO antigens (57). This property may explain why cryopreserved allografts can function long-term without HLA or ABO matching in the absence of immunosuppression. Even in the setting of cardiac transplant rejection, the valves often remain unaffected, suggesting an immunological distinction from the surrounding myocardial tissue (57). Recognising this phenomenon has important implications for tissue-engineered heart valves, since it raises the prospect of developing constructs that minimise immunogenicity while maintaining long-term functionality.

## 5 Conclusion

Our understanding of heart valves has advanced significantly, revealing that these structures are far more complex and active than previously thought. The diverse roles of VECs and VICs in maintaining valve integrity, their dynamic interactions, and the implications of endothelial-to-mesenchymal transformation in disease progression underscore the need for more sophisticated therapeutic approaches. Identifying various VIC phenotypes and their contributions to physiological and pathological processes, such as calcification, emphasises the potential for regenerative therapies targeting these cells. Moreover, the emerging recognition of the nervous system's influence on valve function opens new avenues for innovative treatments. Given the limitations of current surgical interventions, which often rely on passive mechanical replacements, a paradigm shift towards approaches that incorporate the dynamic biological functions of heart valves is imperative. Tissue-engineered heart valves, designed to replicate the native structure and function, represent a promising direction for future research and clinical application. A holistic understanding of heart valves, integrating cellular, molecular, and neural perspectives is needed in the future. This comprehensive approach will pave the way for innovative treatments and significantly enhance the management of valvular heart disease.
